# Mechanically excellent nacre-inspired protective steel-concrete composite against hypervelocity impacts

**DOI:** 10.1038/s41598-021-01308-0

**Published:** 2021-11-09

**Authors:** Yong Mei, Jinming Liu, Yuan Cui, Feng Li, Xuke Tang, Miao Sun, Ruiqiang Chi, Yongbo Zhang, Ao Zhang, Ke Chen

**Affiliations:** 1 Institute of Defense Engineering, AMS, PLA, Beijing, 100036 China; 2 Beijing Advanced Innovation Center for Biomedical Engineering, Key Laboratory of Bio-Inspired Smart Interfacial Science and Technology of Ministry of Education, School of Chemistry, Beihang University (BUAA), Beijing, 100191 China; 3 School of Aeronautic Science and Engineering, Beihang University (BUAA), Beijing, 100191 China; 4 State Key Laboratory of Explosion Science and Technology, Beijing Institute of Technology, Beijing, 100081 China; 5 Hypervelocity Impact Research Center, Harbin Institute of Technology, Harbin, 150080 China

**Keywords:** Mechanical engineering, Composites, Mechanical properties

## Abstract

Steel–concrete (SC) composite widely used in military defensive project is due to its impressive mechanical properties, long-lived service, and low cost. However, the growing use of hypervelocity kinetic weapons in the present war puts forward higher requirements for the anti-explosion and penetration performance of military protection engineering. Here, inspired by the special ‘brick-and-mortar’ (BM) structural feature of natural nacre, we successfully construct a nacre-inspired steel–concrete (NISC) engineering composite with 2510 kg/m^3^, possessing nacre-like lamellar architecture via a bottom-up assembling technique. The NISC engineering composite exhibits nacreous BM structural similarity, high compressive strength of 68.5 MPa, compress modulus of 42.0 GPa, Mohs hardness of 5.5, Young’s modulus of 41.5 GPa, and shear modulus of 18.4 GPa, higher than pure concrete. More interestingly, the hypervelocity impact tests reveal the penetration capability of our NISC target material is obviously stronger than that of pure concrete, enhanced up to about 46.8% at the striking velocity of 1 km/s and approximately 30.9% at the striking velocity of 2 km/s, respectively, by examining the damages of targets, the trajectories, penetration depths, and residual projectiles. This mechanically integrated enhancement can be attributed to the nacre-like BM structural architecture derived from assembling the special steel-bar array frame-reinforced concrete platelets. This study highlights a key role of nacre-like structure design in promoting the enhanced hypervelocity impact resistance of steel–concrete composites.

## Introduction

Concrete composite as one of inorganic materials has key role to society, including in civil building construction, mechanical engineering, and military facilities (*e.g.*, bulletproof walls and structure in nuclear power facilities, and the protective shelters of surface facilities) owing to its excellent formability, fire resistance, and durability^1–3^. However, with the gradual emergence of variously advanced offensive hypersonic weapons (the striking velocity reaches possible up to 2 km/s), the penetration capability and destructive effect of projectiles have been greatly improved. Notably, the impact resistance of traditional reinforced concrete structures such as steel–concrete armours has been not enough to satisfy with the protective requirements of these critical facilities^4–6^. Therefore, the exploitation of advanced protective steel–concrete (SC) materials and structures with light weight, robust mechanical performance, and excellent anti-penetration capability has become an important research field^5,7^.

In nature, numerous shock-resistant biomaterials such as mollusk shells, bone, and mammalian tooth enamel comprise primarily some inorganic substances and are used for structural purposes^8–12^. Among various types of biogenic forms of biominerals, the most popular example is probably mollusk shell with nacreous microstructural feature, where a ‘brick-and-mortar’ (BM) structure in nacreous layer comprised of 95 vol.% of platelets of inorganic aragonite (CaCO_3_) and 5 vol.% of a protein^13–15^. The impressive fracture toughness of natural nacre makes it one of the most widely studied biomaterials which can achieve a toughness, orders of magnitude greater than either of its constituents^8,16^, and meanwhile, the BM design strategy is a unique inspiration for numerous material scientists. Many researchers’ groups have intelligently utilized the strategy to prepare various nacre-like hybrid composites including long fiber, thin films, and centimeter-sized bulk materials, which even obtain more outstanding mechanical properties compare with those of the nacreous layer^17–22^. Therefore, by understanding this advantage in the fascinating spatial structural heterogeneity of natural nacre, it will guide some new high-performance engineering composite materials which can be potentially applied in military facilities and civil building engineering. However, in spite of two decades of research effort, this nacre-inspired strategy mainly focuses on the preparation of small (centimeter)-sized composite materials^19,22,23^. Obviously, large-sized, nacre-inspired composite bulks have wider practical applications in military facilities in comparisons with small-sized composites. With respect to concrete composite materials, although a number of protective SC composite barriers with various structural features have been developed in previous reported works^4–7,24–26^, there is no example using this nacre-inspired designed strategy to create high anti-penetration performance SC composite architectures.

In this work, inspired by the BM structural feature of natural nacre, we successfully construct large-sized, protective nacre-like steel–concrete (NISC) composite targets with unique macroscopic lamellar architectures. Our approach is based on the precise controlling assembly of steel-bar array frame-reinforced concrete building blocks on a pre-defined process routine to create architected nacreous macrostructure. During the construction process, we solve two critical challenges: developing the mechanically high-performance steel-meshed building blocks and optimizing the enhanced interfacial interactions between the adjacent lamellar building blocks in the NISC structures. Static mechanical tests reveal that our NISC composite materials achieve compressive strength of 68.5 MPa, compress modulus of 42.0 GPa, Mohs hardness of 5.5, Young’s modulus of 41.5 GPa, and shear modulus of 18.4 GPa, greatly higher than the pure concrete. More importantly, based on the hypervelocity impact tests for examining the damages of targets, the trajectories, penetration depths, and residual projectiles, we demonstrate that the anti-penetration capability of our NISC target material is obviously stronger than that of pure concrete, enhanced up to about 46.8% at the striking velocity of 1 km/s and approximately 30.9% at the striking velocity of 2 km/s, respectively. The excellent mechanical combination can be due to the nacre-like BM structural architecture assembled from the special steel-bar array frame-reinforced concrete platelet glued by a soft organic matrix. Predictably, the successful preparation of large-sized, nacre-inspired SC composite materials will guide us to design and construct building construction and facilities satisfying the protective requirements.

## Results

### Design and assembly

**Figure 1:**
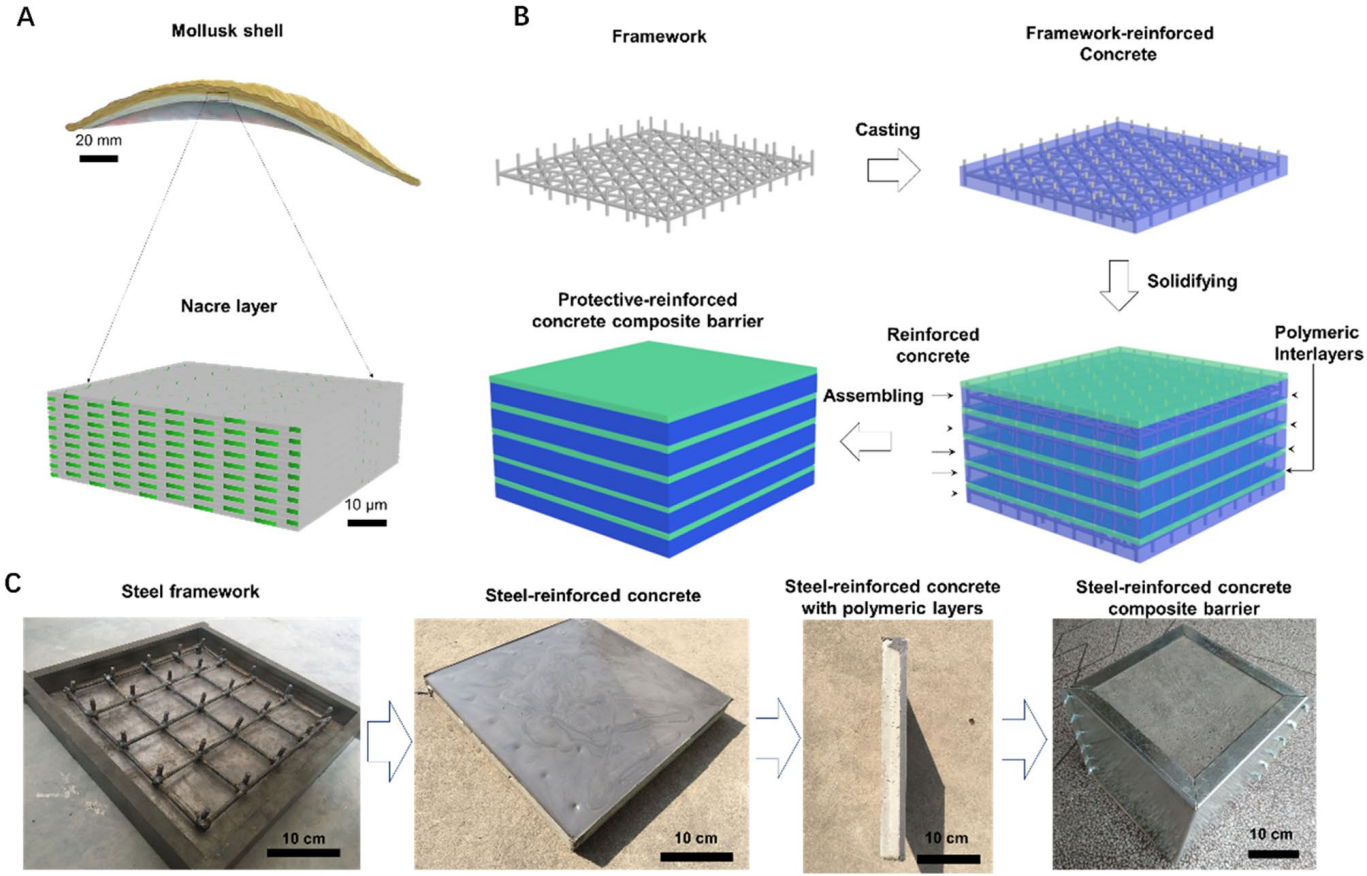
Design and fabrication of nacre-inspired steel–concrete (NISC) composite materials. (**A**) Schematic of multiscale structural features of natural mollusk shell, where nacreous layer made of mineral platelets glued by soft organic matrix, can endow its large deformation, utmost crack limit, and impressive impact energy absorbance by sliding of the micro-platelets. (**B**) Fabrication protocol schematic for the NISC composite bulk targets, referring to framework construction, pouring of concrete, and assembling process. (**C**) Digital photographs for the preparation process of the NISC target material.

Natural nacre material with highly ordered BM microstructure can inspire a new path to construct some advanced engineering materials with outstanding mechanical properties (Fig. 1A and Supplementary Fig. 1), especially excellent impact resistance^27,28^. In this work, we copy nacreous structural features to construct a protective NISC composite material that can embody a higher static mechanical properties and more excellent anti-penetration performance. Firstly, a 5 mm-diameter steel-bar array frame as a reinforced phase prepared by the welding process, consisting of sixteen quadrates (7 × 7 cm) on a plane and a rebar array consisted from sixteen steel bars (30 mm height) located at solder junction perpendicular to the frame plane, as shown in Fig. 1B. The special skeleton structural design can be very efficient to resist large deformation and withstand considerable impact without damage. Secondly, a steel-bar frame is placed into a regular shuttering blended with concrete placing, to construct steel-bar array frame reinforced concrete (SBRC) platelet as an ideal building block (length × width × height (Dimension) = 300 mm × 300 mm × 50 mm), by mimicking nacreous aragonite platelet. The optimal dimension is chosen to create the SBRC platelet with an aspect ratio in the order of 6, which is close to the mineral platelets (6 ~ 10) in natural nacre^8,16^. Thirdly, a ~ 5 mm polyurethane polymeric layer is coated on the surface of the steel-bar frame reinforced concrete platelet to form the polymer-coated steel–concrete structural unit. Noticeably, the thickness of nacreous platelet is approximately 0.3–0.5 μm and the deposition of a 20–30 nm protein layer is intercalated aragonite platelets^13,16^. Therefore, we designed that the thickness ratio of the SBRC platelet to the polymer layer was 10:1 based on the best optimized thickness ratio of aragonite platelet to organic layer in natural nacre. An important step is to select the polymers with mechanical attributes similar to the interfaces in natural mollusk shell. Several polymers such as methyl silicone resin and polyethylene we tested in tension are too brittle or weak (leading to poor energy absorption) (Supplementary Fig. 2A–D). Polyurethane (PU) polymer composite material is eventually selected as the cross-linked interface layer because of its relatively high strength (approximately 30 MPa), very high deformability (about 32%) in tension, strain hardening, and high energy absorption (Supplementary Fig. 2E, F). Fourthly, several SBRC platelets are orderly stacked or laminated with ~ 5 mm thick polymeric interlayers. During the assembly, the SBRC platelets are carefully aligned so that they formed a staggered nacre-like arrangement^13^. Finally, two kinds of NISC composite target materials (Dimension = 300 mm × 300 mm × 100 mm and 300 mm × 300 mm × 300 mm), are successfully obtained by the above assembly technique, named by NISC-100 and NISC-300, respectively.

The whole preparation process of the NISC composite material is simply shown in Fig. 1C (from left to right): I) the unique steel-bar frame was finely welded; II) the concrete was poured into the framework to form the SBRC platelet; III) the 5 mm-thickness polymer material was uniformly coated on the surface of the steel–concrete structural unit; IV) the NISC composite material was finally constructed to be applied as test target material, For the convenience of subsequent performance testing, these target materials were packaged by home-made galvanized iron molds (see detailed in Supplementary Fig. 3). Based on the above structural optimization, we consider that, the effect of macroscopic nacre-like architectural design has probably a superior advantage in enhancing static and dynamic mechanical properties of SC composite compared with conventional homogeneous arrangement. This designed superiority has been resoundingly demonstrated by the construction of the impact-resistant nacre-like super glass composite^27^.

### Static mechanical performance

**Figure 2:**
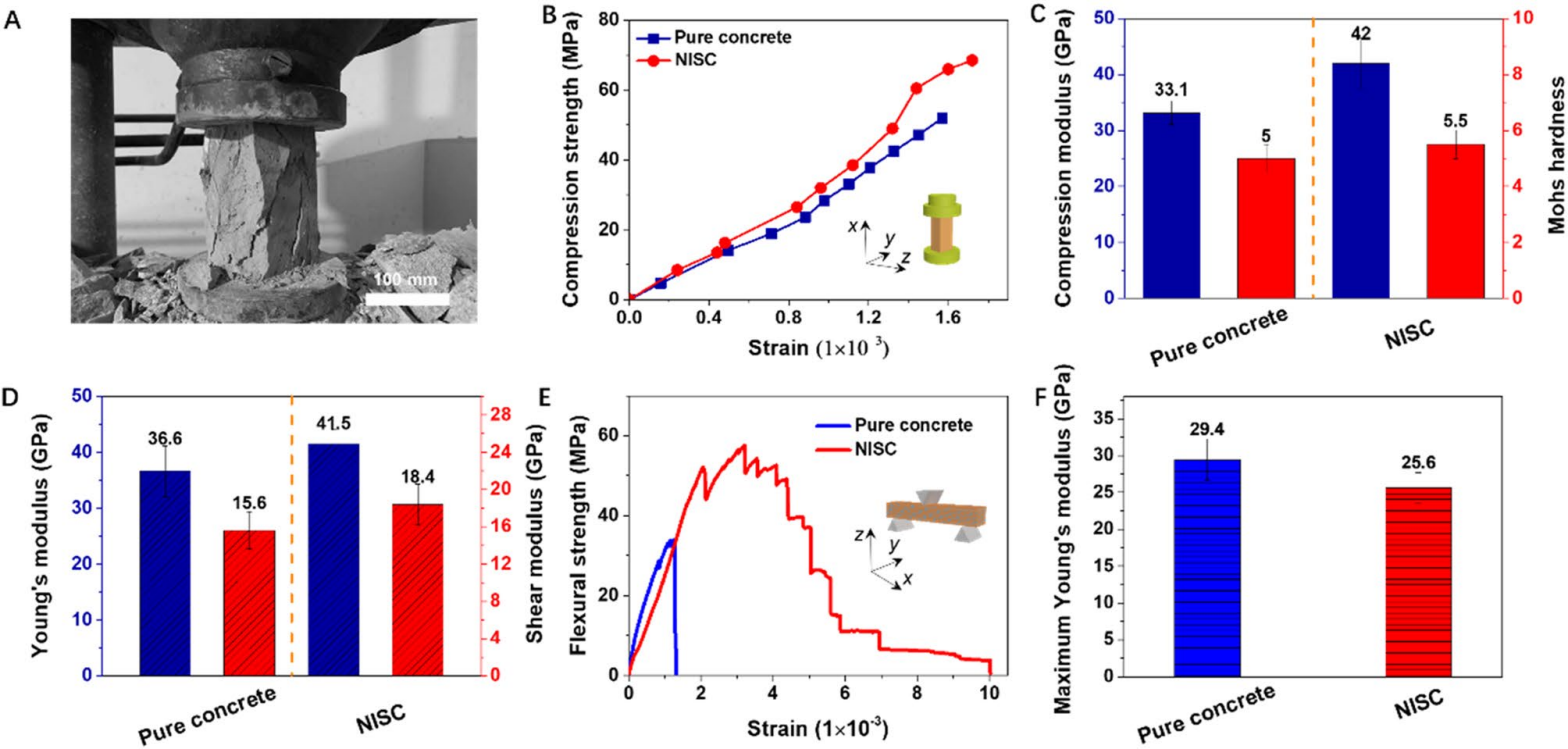
Comparison for the static mechanical properties of the pure concrete and NISC target **materials.** (**A**) Digital photograph of the typical NISC test specimen on a microcomputer controlled electro-hydraulic servo pressure tester, showing the compression test. (**B**, **C**) Typical compression strength-strain curves of the pure concrete and NISC material (**B**), respectively, corresponding to compression modulus and Mohs hardness (C), derived from the compression tests along the steel–concrete platelet (*x* axis) direction. (**D**) Young’s modulus and shear modulus derived from the bending tests along the platelet (*x* axis) direction. (**E**, **F**) Flexural strength-strain curves of the pure concrete and NISC material derived from the bending tests along the platelet stacking (*z* axis) direction (**E**), corresponding to their maximum Young’s modulus (**F**).

To prove the validity of this nacre-inspired BM design strategy to construct the NISC composite material, the static mechanical responses of the pure concrete and NISC composite materials were systematically studied, as shown in Fig. 2. Before performing the mechanical tests, these materials were divided into small species with special dimensions to satisfy the requirement of static mechanical performances (see Supplementary Table 1). For static compression test, it was observed that the specimen was deformed/broken along the steel–concrete platelet (*x* axis) direction in Fig. 2A. The experimental results indicated that the NISC composite displayed a better compressive mechanical performance in comparison with the pure concrete. The compression strength of the NISC composite was about 68.5 MPa, which was about 32.0% higher than that (51.9 MPa) of pure concrete (Fig. 2B). The values in compression modulus and Mohs hardness of the NISC composite were approximately 42 GPa and 5.5 GPa, respectively, clearly superior to those of pure concrete (Fig. 2C). Three-point bending tests along *x* axis direction revealed that the NISC composite achieved a better bending resistance capability compared with pure concrete. The maximum Young’s modulus and shear modulus of the NISC composite are clearly enhanced, up to 41.5 GPa and 18.4 GPa, respectively, superior to those of the pure concrete (36.6 ± 4.5 GPa and 15.6 ± 2.0 GPa) (Fig. 2D).

In addition, we found that, although flexural strength (about 58.3 MPa) of the NISC composite was distinctly higher than that of pure concrete (approximately 35.2 MPa), its Maximum Young’s modulus (about 25.6 MPa) was slightly lower than that of pure concrete (approximately 29.4 MPa), based on the three-point bending tests along the platelet stacking (*z* axis) direction, as shown in Fig. 2E,F. And the NISC composite occurred nonlinear deformation or its flexural strength gradually reduced when its strain was over 0.002. This damage tolerance phenomenon could be attributed to plastic deformation by the polymeric interlayer in heterogeneous BM structure. Notably, the mechanical responses of the NISC composite were stronger along *x* axis direction than those of pure concrete along *z* axis direction, demonstrating that our NISC material is anisotropic. In this case, the optimal steel–concrete composite achieves an exceptional mechanical combination, which can potentially be utilized as an ideal target material.

### Penetration of projectiles

**Figure 3:**
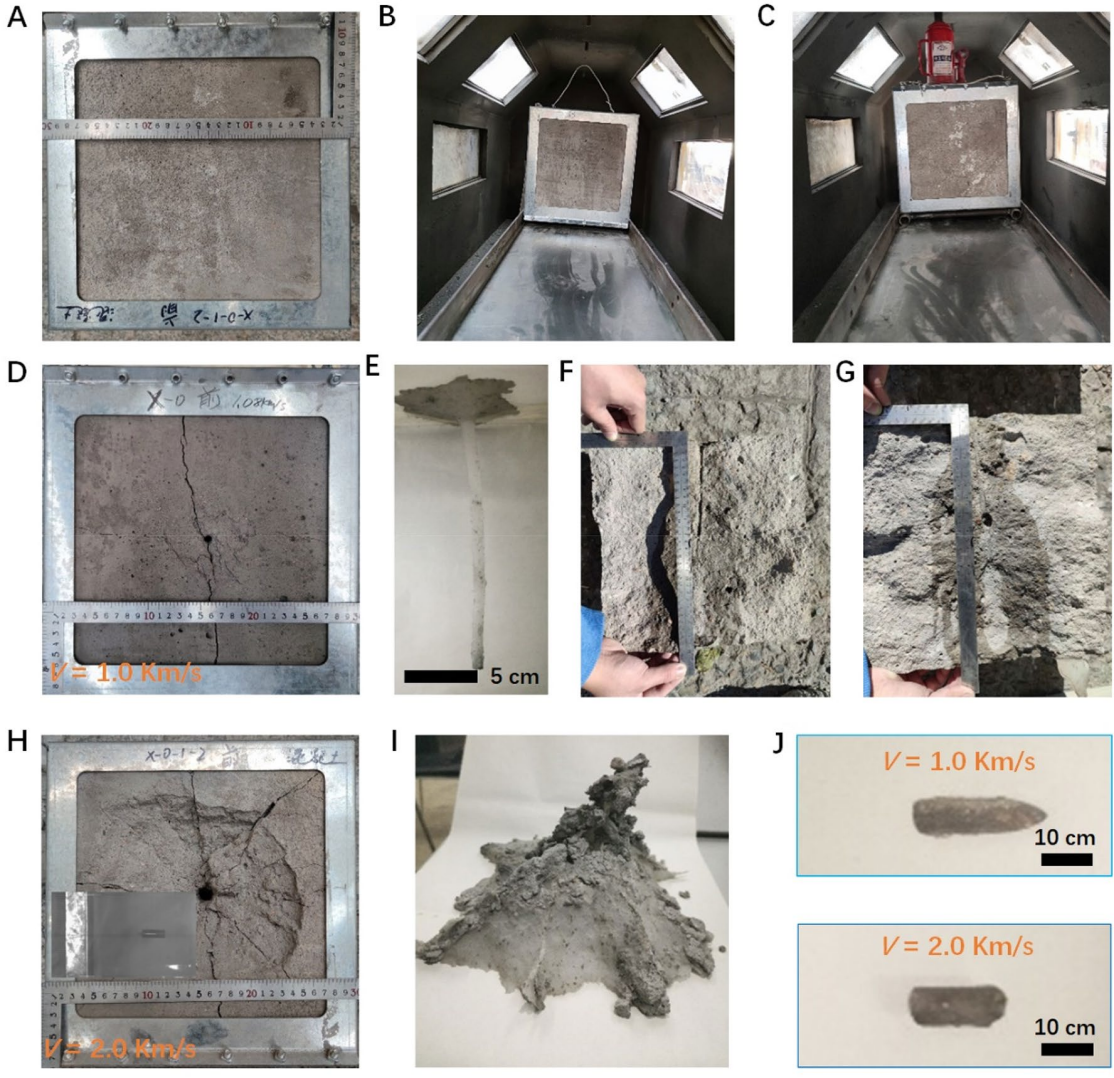
Photographs of typical target materials in the target chamber, their damage observation (including the cratering area, diameter and depth, and trajectory) and eroding projectile, after the hypervelocity impact tests under different striking velocities (*v* = 1 km/s, 2 km/s). (**A**) Photograph of the frontal concrete target. (**B**) Photograph of the frontal concrete target in the target chamber. (**C**) Photograph of the frontal concrete target fixed in the target chamber. (**D**) Photograph of the cratering of the frontal concrete target after a striking velocity of 1 km/s, showing a quasi-linear, long crack path across the bullet hole. (**E**) The j-shaped trajectory in the concrete target at *v* = 1 km/s. (**F**, **G**) Cutting target (**F**) and the sectional view (**G**) of typical concrete target at *v* = 1 km/s. (**H**) The cratering of the frontal concrete target at *v* = 2 km/s, showing that a heavy damage and large cracks occur on the impact surface. (**I**) Photograph of the heavy damaged state within the concrete target at *v* = 2 km/s. (**J**) The morphologies of the residual projectile after the penetration under *v* = 1 km/s (top) and 2 km/s (bottom), respectively.

**Figure 4:**
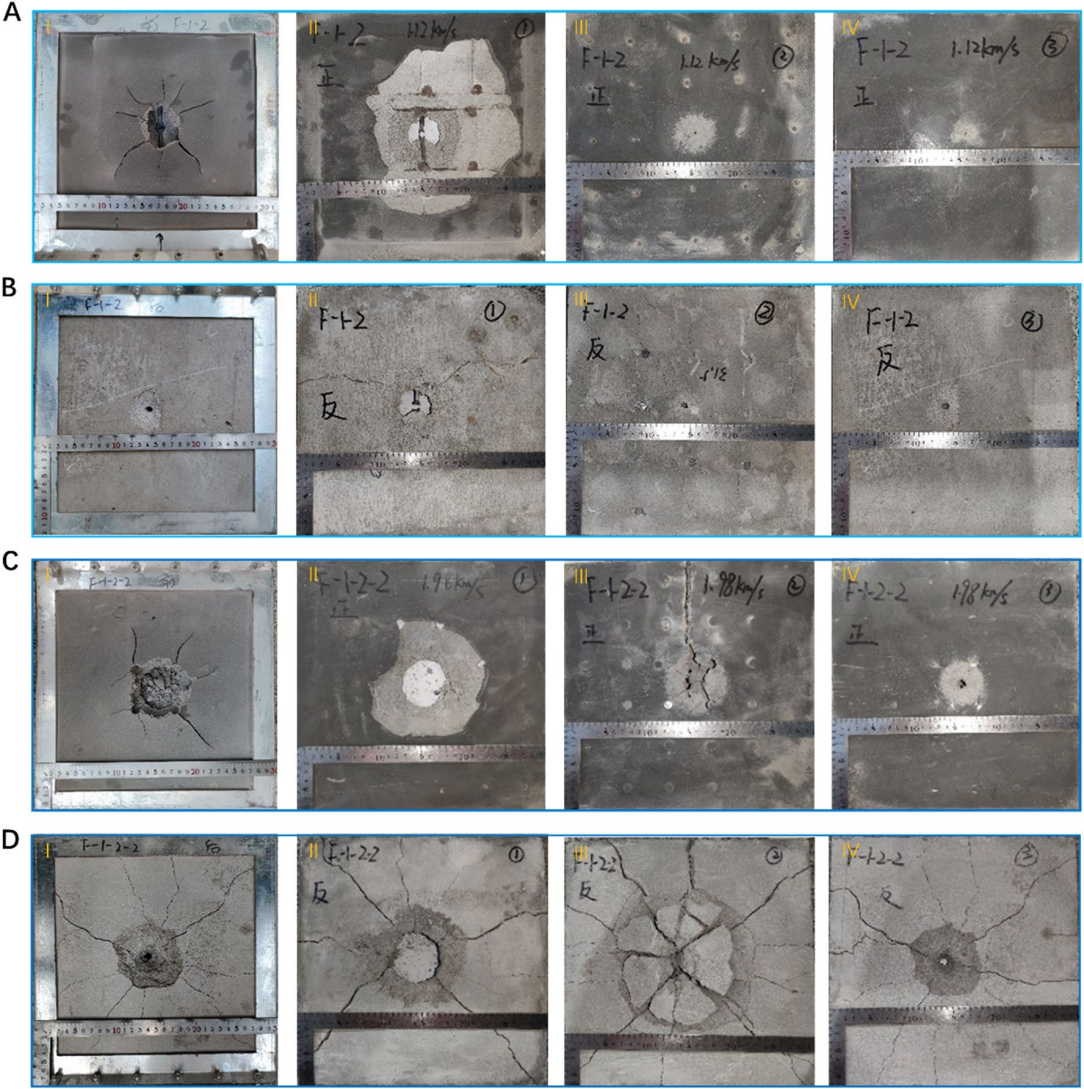
The observation of damage morphologies of the typical NISC-100 targets with different assembled layers, after the hypervelocity impact tests under different striking velocities (*v* = 1 km/s, 2 km/s). (**A**) Morphologies of the surface damage of the frontal NISC target (*v* = 1 km/s). (**B**) Morphologies of the surface damage of the back NISC target (*v* = 1 km/s). (**C**) Morphologies of the surface damage of the frontal NISC target (*v* = 2 km/s). (**D**) Morphologies of the surface damage of the back NISC target (*v* = 2 km/s). From II to IV: the first layer, the second layer, and the third layer.

**Figure 5:**
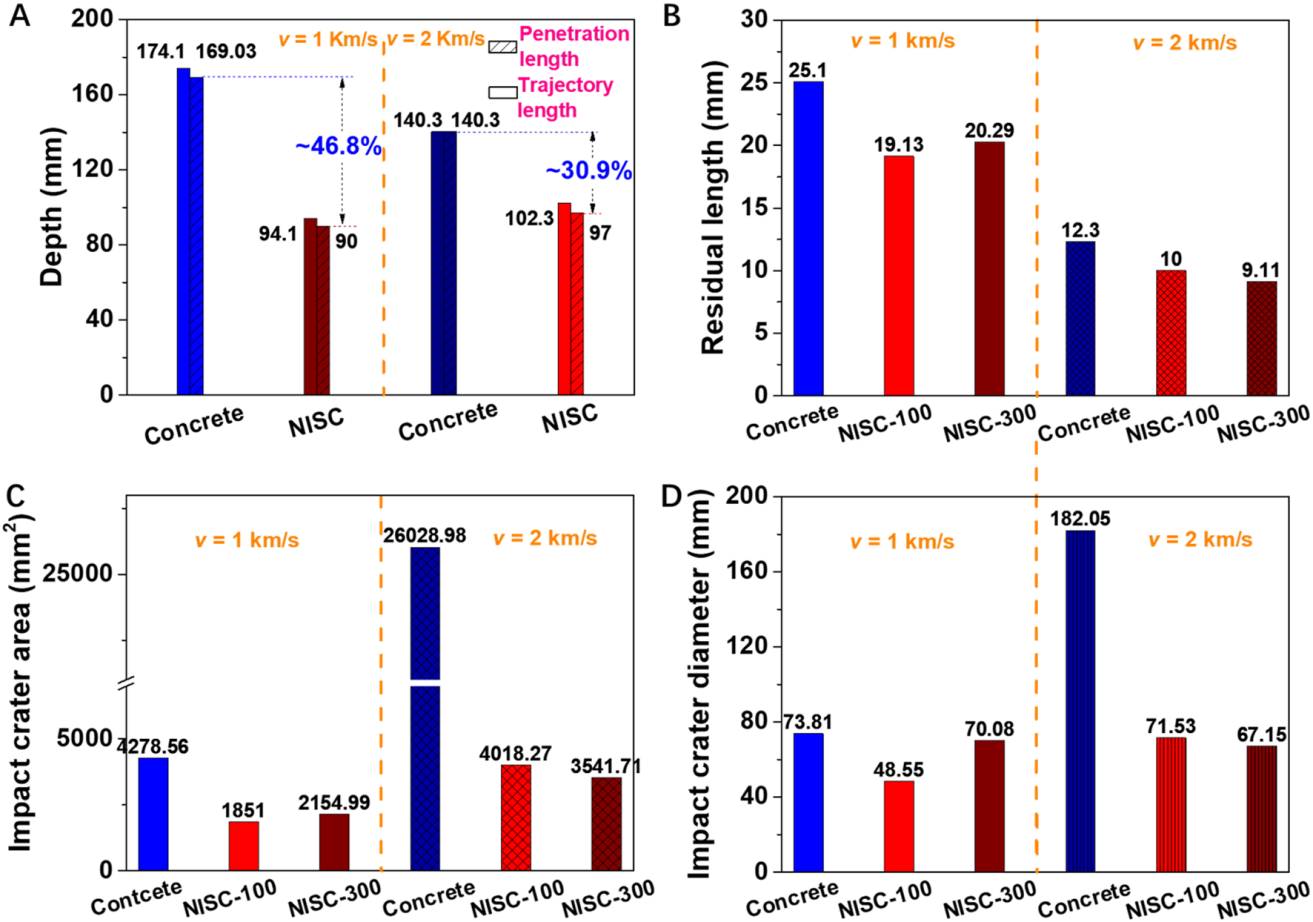
Comparisons for ballistic performance of the pure concrete and typical NISC targets under different striking velocities (1 km/s and 2 km/s). (**A**) Trajectory lengths and penetration depths of the pure concrete and the NISC targets. (**B**) Residual length of the projectile bodies. (**C**) Impact crater areas of the pure concrete, NISC-100, and NISC-300 at their front sides. (**D**) Equivalent diameters of the impact craters of the pure concrete, NISC-100, and NISC-300 at their front sides.

The anti-penetration capabilities of the NISC composite targets are then systematically investigated by the hypervelocity impact tests to compare with that of the pure concrete under different striking velocities (*v* = 1 km/s and 2 km), as shown in Figs. 3, 4, and 5, Supplementary Figs. 5–9 and Movies 1–3. Several shots of pointy ovoid-head steel projectiles (30CrMnSiA) penetrating into the pure concrete and NISC targets were conducted. Firstly, the hypervelocity impact impressions on these target materials are observed with optical photographs and Movies (Figs. 3 and 4, and Supplementary Movies 1–3). Before the shots, the intact structural state of the target in the test equipment is shown in Fig. 3A–C. After the shots under a striking velocity of 1 km/s, there was a about 5-mm bullet hole in the pure concrete. And a crack propagation along the bullet hole (the long crack cut through the whole target) to generate and the surficial concrete around the bullet hole also isn’t destroyed, as displayed in Fig. 3D. Obviously, the localized damages occurred on the impact surfaces of the concrete target, confirming that the dimensions of the target are large enough to neglect the boundary influences^29^. To capture the shape and dimensions of the impact craters more precisely, the silicone casting method was used to reconstruct the 3D crater and the projectile trajectory within the pure concrete, as shown in Fig. 3E. The damaged state of the pure concrete target, dimensions of crater, and a *j*-shaped crater followed by a cylindrical tunnel could be clearly observed. And then, typical concrete targets were carefully cut along the penetration boreholes, the sectional views of the typical concrete targets are exhibited in Fig. 3F,G. We could observe that the projectile penetration formed a frustum-shaped crater followed by a cylindrical tunnel. As we all know, the diameter of tunneling is consistent to that of projectile when the striking velocities are relatively low where the projectiles can be considered as rigid bodies^30^. However, when the striking velocity was enhanced up to 2 km/s, the bullet hole in the pure concrete was obviously broaden, up to about 15 mm. Clearly, the tunneling diameter under was larger than that of the projectile, confirming the assumptions made by several researcher’s groups^29,30^. Several large cracks propagated around the bullet hole (these large cracks passed through the whole target), a large area of the surficial concrete around the bullet hole was seriously destroyed. And a heavy damaged state could be observed within the concrete target. These results verified that it wasn’t unaffordable for the common concrete against hypervelocity impacts.

Furthermore, after the impact tests, the pure concrete target was carefully cut to recover the residual projectile. Figure 3J presents the photographs of the two recovered projectiles (*v*_0_ = 1 km/s (top) and *v*_0_ = 2 km/s (bottom)). Obviously, compared to the unfired projectile, the length and mass of residual projectile reduced a greater or lesser degree. The residual length and mass of 1 km/s recovered projectile (*l*_1_, *m*_1_) and 2 km/s recovered projectile (*l*_2_, *m*_2_) are 25.1 mm/5.48 g and 12.3 mm/3.56 g (see Table 1). The residual length and mass loss of the projectile at a striking speed of 2 km/s were much more serious than that at a striking velocity of 1 km/s. Quantitatively, the residual length and mass loss of the projectile changed from 26.4 mm/5.76 g for unfired projectile to 25.1 mm/5.48 g for 1 km/s recovered projectile to 12.3 mm/3.56 g for 2 km/s recovered projectile, respectively. Correspondingly, when the initial velocity of the projectile was 1 km/s, the deformation of the projectile was no evident, as shown in Fig. 3J. The blunted length of the recovered projectiles was only 4.9%, while the relative mass loss was also 4.9%. This regime indicated that the projectile was sightly deformed but not eroded during the penetration process. When the initial velocity of projectile was enhanced up to 2 km/s, the relative mass loss was distinctly exacerbated and reached 38.2%. The mass loss and residual length of projectile increased and decreased with the rising of striking velocity, respectively. This regime was considered as eroding projectile penetration.

**Table 1:** Comparison for experimental results on different types of typical target materials after hypervelocity impacts.

Target names	Density (kg/m^3^)	Actual velocity (km/s)	Residual length (mm)	Residual mass (g)	Trajectory length (mm)	Penetration depth (mm)	Impact crater pixels (N)	Impact crater area (mm^2^)	Equivalent diameter of impact crater (mm)
Concrete	2410	1.080	25.10	5.48	174.10	169.03	814,809	4278.56	73.81
Concrete	2410	1.937	12.30	3.56	140.30	140.30	4,873,163	26,028.98	182.05
NISC-100	2510	1.118	19.13	4.94	100.00	–	352,504*	1851.00*	48.55*
NISC-100	2510	1.984	10.00	2.96	100.00	–	765,239*	4018.27*	71.53*
NISC-300	2510	1.089	20.29	5.01	94.100	90.00	410,396*	2154.99*	70.08*
NISC-300	2510	2.000	9.11	2.72	102.30	97.00	674,483*	3541.71*	67.15*

*The first layer of the NISC target material.

In contrast, we found that the impact impressions of the NISC composite target were clearly distinguished from that of pure concrete. After the NISC composite target was impacted under a striking velocity of 1 km/s, a projectile passed through the steel bar on the front NISC-100 target, leaving a small bullet hole in the pure concrete and a small crater; and a small crack propagation around the bullet hole. But these short cracks could not cut through the NISC target, and the surficial concrete around the bullet hole also was slightly damaged (Fig. 4AI). In order to further study the damaged state inside the NISC-100 target at a striking velocity of 1 km/s, we observed the different layers (from II to IV), as shown in Fig. 4AII–IV. In the first layer, a small region on the surface of the concrete was peeled off. However, the damaged area gradually reduced from the second to third layer. On the back of the NISC-100 target (Fig. 4BI–IV), we only observed a tiny piece of concrete around the bullet hole. The damaged area had no obvious difference among these assembled layers.

However, after shooting, the bullet hole in the front NISC-100 target at a striking speed of 2 km/s became obviously larger than that at a striking velocity of 1 km/s. And several small cracks propagated around the bullet hole (these small cracks could not cut through the NISC target). Also, a ~ 5 cm^2^ area of the surficial concrete around the bullet hole is destroyed, as shown in Fig. 4CI. With the number of layers increasing, the damaged area rapidly reduced (Fig. 4CII–IV), clearly lower than that of the pure concrete (Fig. 3). However, at the back of the NISC-100 target (Fig. 4D), a 5 cm^2^ area of surficial concrete around the bullet hole is peel off, accompanying with some curved crack propagation paths around the bullet hole (Fig. 4D–I). Although the crack could cut through the NISC target (see the different assembled layers), as shown in Fig. 4DII–IV, the damaged degree of the NISC target was notably smaller than that of the pure concrete. Based on the analysis of these damaged morphologies, we preliminarily demonstrate that our nacre-inspired steel-bar-reinforced concrete lamination clearly enhance the damage tolerance of concrete materials under hypervelocity impacts.

In addition, to quantitatively estimate the impact resistance capability of the bioinspired composite target, some important parameters (such as trajectory depth, penetration depth, residual length and mass of projectiles, impact crater area, and impact crater diameter) are summarized and compared in Fig. 5A–D, Table 1 and Supplementary Fig. 9. Generally, the impact resistance ability of the NISC target material was stronger than that of pure concrete material. For example, at a striking speed of 1 km/s, the trajectory depth, penetration depth, residual length and mass of the projectile, impact crater area, and impact crater diameter of the NISC target materials were 94.1 mm (NISC-300), 90.0 mm (NISC-300), 19.13 mm/4.94 g and 20.29 mm/5.01 g (NISC-100 and NISC-300), 1851/2154.99 mm^2^ (NISC-100 and NISC-300), 48.55/73.08 mm (NISC-100 and NISC-300), respectively, obviously lower than those of the pure concrete (174.1 mm, 169.03 mm, 20.29 mm/5.01 g, 278.56 mm^2^, and 73.81 mm). However, at a striking velocity of 2 km/s, the trajectory depth, penetration depth, residual length and mass of the projectile, impact crater area, and impact crater diameter of the NISC target materials were 102.3 mm (NISC-300), 97 mm (NISC-300), 10/9.11 mm and 2.96/2.72 (NISC-100 and NISC-300), 4018.27/3541.7 mm^2^ (NISC-100/NISC-300), and 71.53/67.15 mm (NISC-100 and NISC-300), respectively, notably lower than those of the pure concrete (140.3 mm, 140.3 mm, 12.3 mm/3.56 g, 26,028.98 mm^2^, 182.05 mm). Simultaneously, we also found that the impact parameters of NISC-300 were lower than those NISC-100, suggesting that with increasing of the thickness of the bioinspired target, the impact resistance capability could be gradually enhanced.

## Discussion

Based on the above static mechanics and anti-penetration capabilities of NISC composites, it is generally confirmed that our nacre-like BM structural design, together with the special steel-bar array frame as a reinforced phase, are synergistically conducive to further enhance the load-bearing and impact resistance capabilities of a concrete material. As for static mechanical tests, compared with the homogeneous structure of the pure concrete, on the one hand, the soft interlayer design generates larger nonlinear deformations over large volumes and show higher fracture toughness, on the other hand, the steel-bar array frame in concrete dramatically achieves higher strength and modulus/stiffness. Therefore, the coeffect of the BM structural design and steel-bar array frame results in the impressive mechanical properties of our NISC composites. Similarly, in terms of the impact tests, the high-performance SBRC platelet resists the hypervelocity impact, in other word, the damage of the one-dimensional impact is mostly confined surrounding steel-bar array frame of the concrete target, and the soft interlayer efficiently absorb the impact energy^29,31^. Thus, the static mechanical improvement of a material is benefit to enhance its impact resistance capability to a great extent. Besides, we also account for the discontinuity in rebar when assembling the SBRC platelet. For small sized projectile (Ф ≤ 7 mm, near to the rebar diameter), the anti-penetration effect is stronger when hitting the rebar frame than that when hitting the concrete matrix. However, for large sized projectile (Ф > 7), we consider that the discontinuity in rebar of the composite target can be ignored.

In recent years, many researchers’ groups have performed high strength steel (4340 steel, AerMet steel) projectile penetration experiments on concrete targets, in which the strengths of targets of projectiles were ranged from 37.4 MPa to 65.6 MPa, corresponding striking velocities were ranged from 660 to 1225 m/s, respectively^31–33^. These projectiles were demonstrated to be eroded after the penetration tests, showing that the penetration process entered into the eroding stage for these projectiles/target integrations during the above striking velocity ranges^34^. Generally, when the striking velocity of a penetration weapon is relatively low (*e.g.*, less than 0.8 km/s), the projectile deformation and failure can be negligible during the whole penetration process, therefore the penetration capability can be predicted through considering the projectile as a rigid body^29^. However, with respect to our hypervelocity impact tests, the projectile must be blunted and even eroded owing to high pressure generated at the projectile/target interface, resulting in a drastic reduction of projectile penetration performance. Correspondingly, when the initial velocity of projectiles is 1 km/s, the blunted lengths of the recovered projectiles within NISC-100 and NISC-300 are 27.5% and 23.1%, respectively, while the relative mass losses are 14.2% and 13.0%. During the penetration process the projectile must be partially deformed but not eroded. Furthermore, when the initial velocity of projectile is enhanced up to 2 km/s, the blunted lengths and mass loss are visibly exacerbated, reach 62.1% and 65.5%, respectively. The residual length and mass loss of projectile increase and decrease with the rising of striking velocity, respectively. Similarly, during our penetration tests, the projectile occurs a large deformation and serious erosion, which can be considered as eroding projectile penetration. Besides, through comparing penetration depth of pure concrete with that of the NISC target, we reckon that, the penetration resistance capability of the NISC target material can be enhanced up to about ~ 46.8% at the striking velocity of 1 km/s and ~ 30.9% at the striking velocity of 2 km/s, respectively. This large enhancement in the impact resistance of the bioinspired concrete material can be attributed to the co-effect of the special steel-bar framework-reinforced concrete design and nacre-like BM structural architecture.

As for theoretically predicting the penetration depth derived from high-speed projectile, Alekseevskii-Tate (A-T) model, as a 1D modified hydrodynamic model considering of contributions of projectile strength (*Y*_p_) and target resistance (*R*_t_), can be successfully used in the eroding projectile penetrations into metallic targets, where both projectile strength (*Y*_p_) and target resistance (*R*_t_) have explicit analytical expressions^35,36^. In recent years, an extended dynamic spherical cavity expansion model (EDCEM) was presented by introducing a hyperbolic yield criterion and Murnaghan the equation of state (EOS) to successfully be applied to predict the one-dimension resistance of concrete-like materials for the one-dimension eroding projectile penetration problem^29^. However, the eroding penetration model is only validated for concrete-like homogeneous composite materials. For our nacre-like, heterogeneous SC composite materials there is still no available recognized model to predict the penetration depth of projectile under ultra-high velocity impacting tests. Therefore, the eroding penetration model for bioinspired heterogeneous SC composite to ultra-high projectile impact should be developed in the near future.

## Conclusion

By mimicking the special BM structural features of natural nacre in three dimensions and over large volumes, we firstly prepared a large-sized, excellent mechanical performance nacre-like SC composite barrier by assembling the steel-bars array frame-reinforced concrete building blocks glued by a soft polyurethane polymer in a simple bottom-up process. Compared to the homogeneous structure of pure concrete, the multiscale, hierarchical BM structural design with abundant hybrid interfaces can absorb larger amounts of mechanical energy, providing the composite barrier with higher strength, higher modulus, more excellent impact resistance, and stronger penetration resistance capability. The nacre-like SC composites also depict an architecture with relatively large size but with high order and periodicity, which is fully different from other previously reported nacre-like micro-nanocomposites^17–20,24^. More importantly, the macroscopic lamellar architecture and lamination fabrication method are low-cost and relatively easy to implement into the large-scale production of impact-resistant nacre-like SC composite barriers for a wide range of potential applications, including civil building construction, mechanical engineering, and military facilities.

## Materials and methods

### Materials and processing equipment

Mollusk shell such as red abalone and scallop were purchased from a formal seafood market. Silicate cement (PI52.5) was purchased from Xi’an Qinling Cement Factory. Silver sand (1.6–2.2 mm), high performance water-reducing agents, galvanized iron sheet (Thickness = 1.5 mm), steel bar (R = 8 mm) were purchased from building material market. Methyl silicone resin and curing agent were purchased from Hubei Xinsihai Chemical Co. LTD. Polyethylene (PE) were purchased from Aladdin Chemical Co. LTD. Polyurethane (PU) and curing agent were purchased from Shenzhen Youruiqi Technology Co. LTD. Besides, concrete mixers, cutting machines, forklifts, electric welding, and custom plastic molds were also used to prepare these target materials.

### Experimental procedures

To prepare nacre-like steel concrete composite target, we firstly constructed the steel-bars array frame (Size: 280 mm × 280 mm × 25 mm), which is composed of sixteen quadrates (7 cm × 7 cm) on a plane and a rebar array consisted from sixteen steel bars (30 mm height) located at solder junction perpendicular to the frame plane. Secondly, the well-mixed concrete (its proportional formula is shown in Table 2) was poured to form the steel-bar framework reinforced concrete structural unit. After the solidification of the concrete, a 5 mm-thickness polymeric binder as soft phase was coated on the surface of the steel-bars array frame reinforced concrete structural unit. Next, they were laminated together by stacking to form laminates with a total of 3–10 layers, being packaged by a home-made galvanized iron mold (see Supplementary Fig. 3A). Finally, the NISC composite targets with different sizes (300 mm × 300 mm × 100 mm and 300 mm × 300 mm × 100 mm) were successfully constructed by the bottom-up assembly method (Supplementary Fig. 3B,C). And three polymer films with about ~ 1.0 mm thickness such as methyl silicone resin, PE and PU were also prepared by the direct casting method (Supplementary Fig. 2). For comparison, pure concrete target with similar proportional composition was prepared by the direct casting method (Supplementary Fig. 3D, E).

**Table 2:** The mix proportion of the concrete material.

Target	Content (ratio)			
	Cement	Sand	Aggregate	Water
Concrete	1.1	1	2.72	0.38

### Static mechanical tests

In the static mechanical test, the compressive tests of these target materials were performed on a microcomputer controlled electro-hydraulic servo pressure tester. The testing method is in accordance with the standard of ‘test methods for mechanical properties of ordinary concrete’ (GB/T50081-2002). The working size of the specimens is shown in Table S1 and Supplementary Fig. 4. The bending mechanical tests of specimens were performed on a multifunctional electro-hydraulic servo testing machine. The testing method referred to the standard of test methods for steel fiber reinforced concrete (JG/T472-2015). The tensile tests of these polymer materials were conducted using a Shimadzu AGS-X Tester with a loading speed of 1 mm/min and a sample gauge length of 10 mm. All the films for the tensile tests were cut into strips of length 30 mm and width 7 mm.

### Hypervelocity impact tests

**Figure 6:**
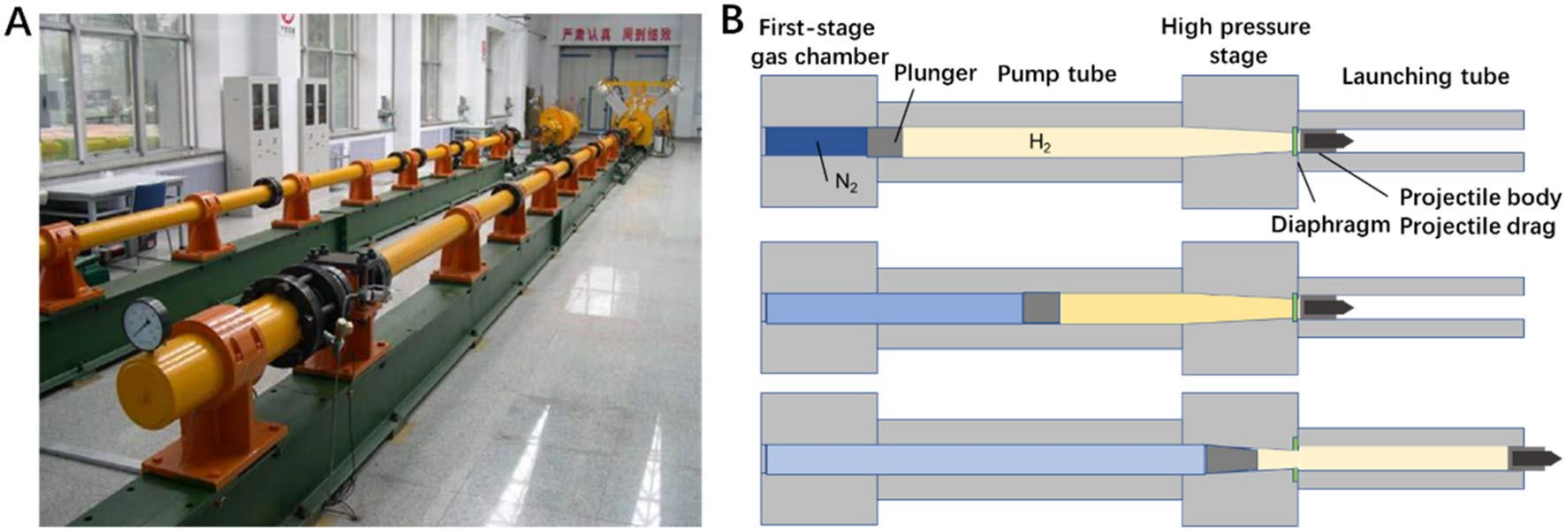
Photograph and working principle of the two-stage light-gas gun. (**A**) The two-stage light-gas gun system. (**B**) The working principle of the two-stage light-gas gun.

As shown in Fig. 6, a two-stage light-gas gun system was employed to launch the projectiles. The system is mainly composed of a launching unit and a vacuum target chamber, as shown in Fig. 6A. The launching unit is used to accelerate the projectile to the target speed and launch projectile into the vacuum target chamber. The vacuum target chamber is used to place the target, in which projectile impacts the target. The diameters of the pump tube and the launching tube are 50 mm and 20 mm, respectively. Furthermore, its working principle is shown in Fig. 6B. Firstly, the N_2_ gas is pushed into the sealed vessel by a compressor until to a designed pressure. Next, the value of N_2_ vessel is opened and the plunger is accelerated in the pump tube which is filled with high-pressure hydrogen gas. At the end of the first-stage pump tube is a conical section, leading down to the second-stage 20 mm caliber launching tube that fires the projectiles. In this conical section, there is a rupture disk with a “ + ” pattern scored into the middle surface. When the hydrogen develops sufficient pressure to burst the scored section of rupture disk, the hydrogen flows through the hole and accelerates the projectiles with sabots located in the second-stage launching tube. After the projectiles are pulled out of the launching tube, the sabots are separated from the projectiles aerodynamically in the separation chamber filled with nitrogen gas (see some detailed process in SI)).

In the target chamber, the targets are placed with their impact surfaces perpendicular to the gun barrel. The striking velocity *v* is measured from a Photron SA5 high-speed camera placed in the projectile trajectory, as shown in Supplementary Fig. 5A. Before shooting, the trajectory position should be calibrated, and a moving distance in the image is obtained according to the actual calibration ruler length. The specific setup parameters are the aperture of 2,100,000 FPS and each frame of 1 μs. The calibration image is exhibited in Supplementary Fig. 5B.

**Figure 7:**
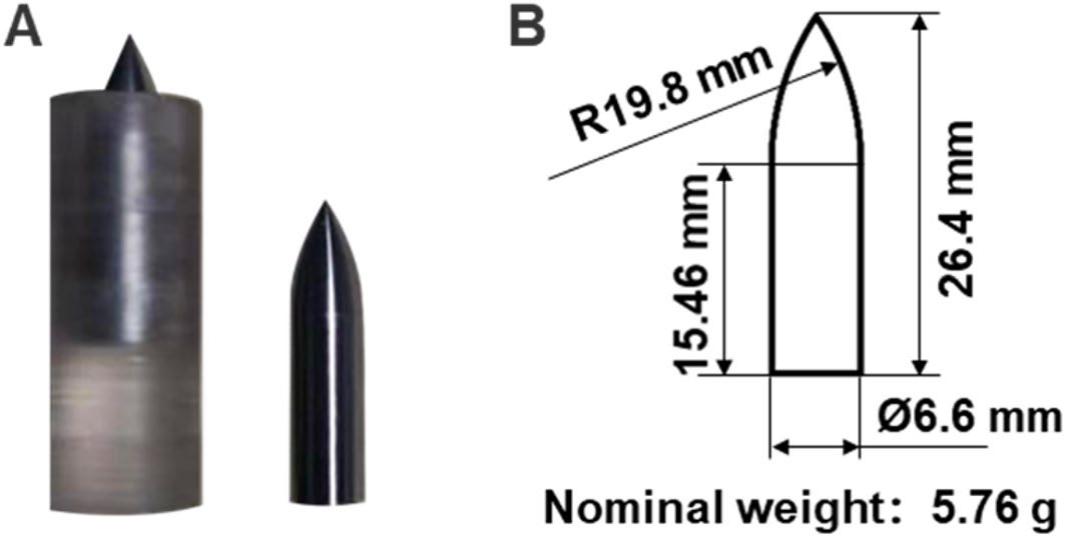
Photograph and dimension parameters of unfired projectile with sabots used in the hypervelocity impact tests. (**A**) Photograph of the unfired projectile with sabots. (**B**) The dimension parameters of the unfired projectile.

The projectile with sabots and its dimension parameter are shown in Fig. 7. The projectile is 30CrMnSiA, with a nominal density of 7.85 g/cm^3^, machining tolerance ± 0.01 mm, and staining treatment. During shooting at a striking velocity of 2 km/s, owing to the relatively large mass of the projectile and the insufficient carrying capacity of the sabots (the elastomer), the projectile would reverse attack the projectile during firing, resulting in substandard incident velocity and even scratching the gun tube. Therefore, a 2 mm × 10 mm titanium alloy gasket is added to reduce the damage of the elastomer. The diameter of the elastomer is 12.65–12.75 mm, so that the total mass of the whole projectile is slightly increased.

According to the condition of normal impact, upper support and rear support are provided for the relatively thick target materials, as shown in Supplementary Fig. 6A. The support doesn’t provide prestress, but only plays a fixing role to prevent the target from turning over or moving. Besides, for testing and observing the thin target with thickness of 100 mm, we installed a sighting board and a protective shield, as shown in Supplementary Fig. 6B. Owing to the large mass of the sighting board and protective shield, no additional support is used.

## Supplementary Information

Supplementary Video 1.

Supplementary Video 2.

Supplementary Video 3.

Supplementary Information.

## Data Availability

All data generated or analysed during this study are included in this published article (and its supplementary information files).
