# Supplementary material for: Mechanically excellent nacre-inspired protective steel-concrete composite against hypervelocity impacts
Source: Sci Rep. 2021 Nov 9;11:21930. doi: 10.1038/s41598-021-01308-0 (PMC8578474; doi:10.1038/s41598-021-01308-0)
Supplement: Supplementary file 4 — Supplementary Information. [file 41598_2021_1308_MOESM4_ESM.docx]

Supplementary Information

**Mechanically Excellent Nacre-inspired** **Protective Steel-Concrete Composite against** **Hypervelocity Impacts**

Yong Mei^1,4,†^, Jinming Liu^1^, Yuan Cui^1^, Feng Li^1^, Xuke Tang^2,†^, Miao Sun^5^, Ruiqiang Chi^5^, Yongbo Zhang^3,^*, Ao Zhang^1,^*, Ke Chen^2,^*

*^1^Institute of Defense Engineering, AMS, PLA, Beijing, 100036, China*

*^2^Beijing Advanced Innovation Center for Biomedical Engineering, Key Laboratory of Bio-inspired Smart Interfacial Science and Technology of Ministry of Education, School of Chemistry, Beihang University (BUAA), Beijing, 100191, China*

*^3^School of Aeronautic Science and Engineering, Beihang University (BUAA), Beijing 100191, China*

*^4^State Key Laboratory of Explosion Science and Technology, Beijing Institute of Technology, Beijing 100081, China*

*^5^Hypervelocity Impact Research Center, Harbin Institute of Technology, Harbin 150080, China*

*Corresponding author

Email: [chenke0119@buaa.edu.cn](mailto:chenke0119@buaa.edu.cn), [zhangyongbo@buaa.edu.cn](mailto:zhangyongbo@buaa.edu.cn), zhangao202012@163.com.

This PDF file includes:

Supplementary Text

Supplementary Figs. 1 to 9

Supplementary Tables 1 and 2

Captions for Supplementary Movies 1 to 3

Other Supplementary Material for this manuscript includes the following:

**The working principle of a two-stage light-gas gun system**

The working principle of a two-stage light-gas gun is briefly introduced as following: i) high-pressure N_2_ gas firstly fills into high-pressure first-stage gas chamber; ii) the start button releases the high-pressure N_2_ to derive the plunger placed in the pump pipe; iii) the plunger accelerates the movement to compress H_2_ gas filled into the pump tube to do work (in this case, H_2_ gas can reach the state of high temperature and high pressure in a short time); iv) high temperature, high pressure H_2_ gas is impacted on the metal diaphragm within a high pressure cone section and causes it to break, and then high temperature and high pressure gas enters the launching tube; v) the projectile is placed in the cartridge case, which are located in the launching tube. After the high-temperature and high-pressure gas enters the launching tube, it will be accelerated; vi) when the projectile is consigned to the exit of the launching tube, the projectile continues to fly along the trajectory and hits the target placed in the vacuum target cabin.

Before shooting, we firstly need to do some preparatory work, such as weighing the projectile and the sabots, inspecting the integrity of the target, lifting the target into the target bin, adjusting the target to ballistic center and high-speed photography area, and checking the trigger switch of high-speed camera. Secondly, we install the projectile after closing the door, connect launch tube with high pressure cone end, install the first-stage piston, and start the vacuum pump of the target chamber. After the vacuum degree of the target chamber is nearly zero, we use N_2_ to fill the first-stage gas chamber up to a setting pressure, and then close the vacuum pump. Thirdly, we close the door after filling a setting pressure of H_2_ in the pump tube, open fill-in light. we record the last shooting stamping value after opening trigger mode of high-speed camera, and then require all employees to leave the lab. Fourthly, we press the launch button and the high-speed camera button. The high-speed photographic images should be saved, and the final target shooting speed is also measured and recorded. Finally, the impacted target is lifted out of the target cabin and saved, the residual projectile is also checked in the target cabin and saved.


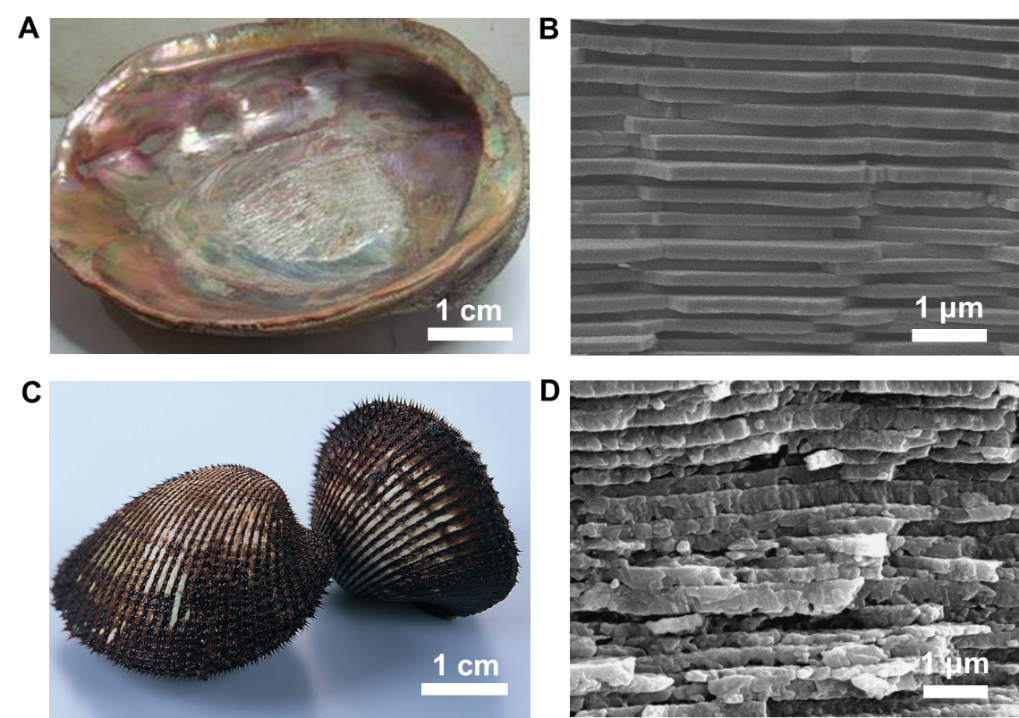


**Supplementary Fig. 1.** [Digital](javascript:;) photos and microstructure features of natural nacre from abalone mollusk (*Haliotis rufescens* and clamshell (*Scapharca subcrenata*. **(A, B)** The photo (A) and scanning electron microscope (SEM) image (B) of the brick-and-mortar structure of natural nacre from abalone mollusk. The photo (C) and scanning electron microscope (SEM) image (D) of the brick-and-mortar structure of natural nacre from clamshell.


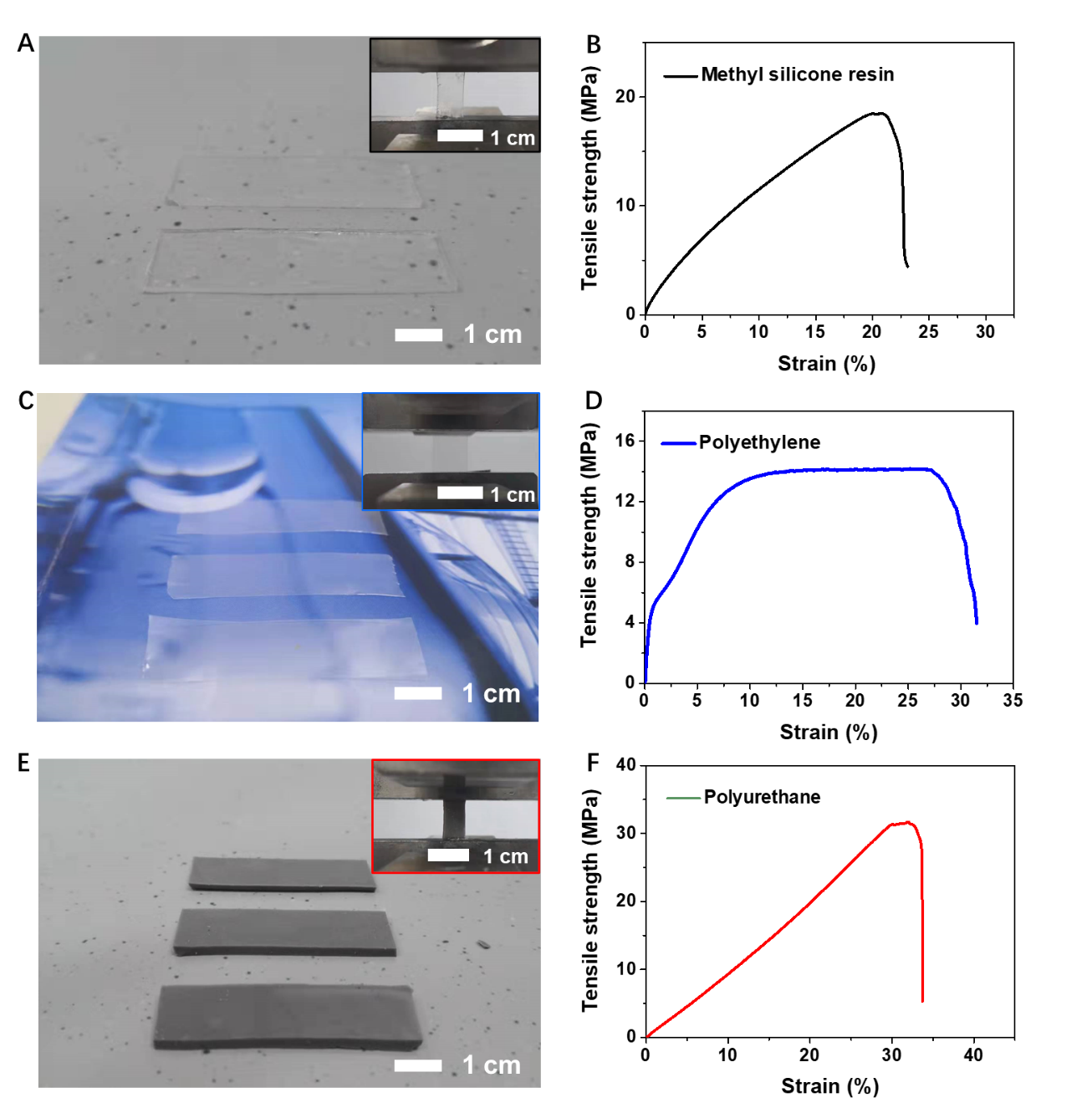


**Supplementary Fig. 2.** Characterization of mechanical properties of three polymer materials. **(A)** Digital photos of the methyl silicone resin film. Inset: the photo of a sample after stretching. **(B)** Tensile strength-strain curve of the methyl silicone resin film. **(C)** Digital photos of the polyethylene (PE) film. Inset: the photo of a sample after stretching. **(D)** Tensile strength-strain curve of the polyethylene film. **(E)** Digital photos of the polyurethane film. Inset: the photo of a sample after stretching. **(F)** Tensile strength-strain curve of the polyurethane film.

To select the proper polymer as the interlayer in the nacre-like composite target, three different polymer films such as methyl silicone resin, PE, and PU were successfully prepared by the casting method and their digital photos and mechanical properties are as shown in Supplementary Fig. 2. Tensile experimental results exhibited that the strengths and strains of methyl silicone resin, PE, and PU were 18.3 MPa/22.0%, 27.0 MPa/29.3%, and 34.1 MPa/34.2%, respectively. Obviously, tensile mechanical properties of PU polymer are clearly higher than those of methyl silicone resin or PE.

**
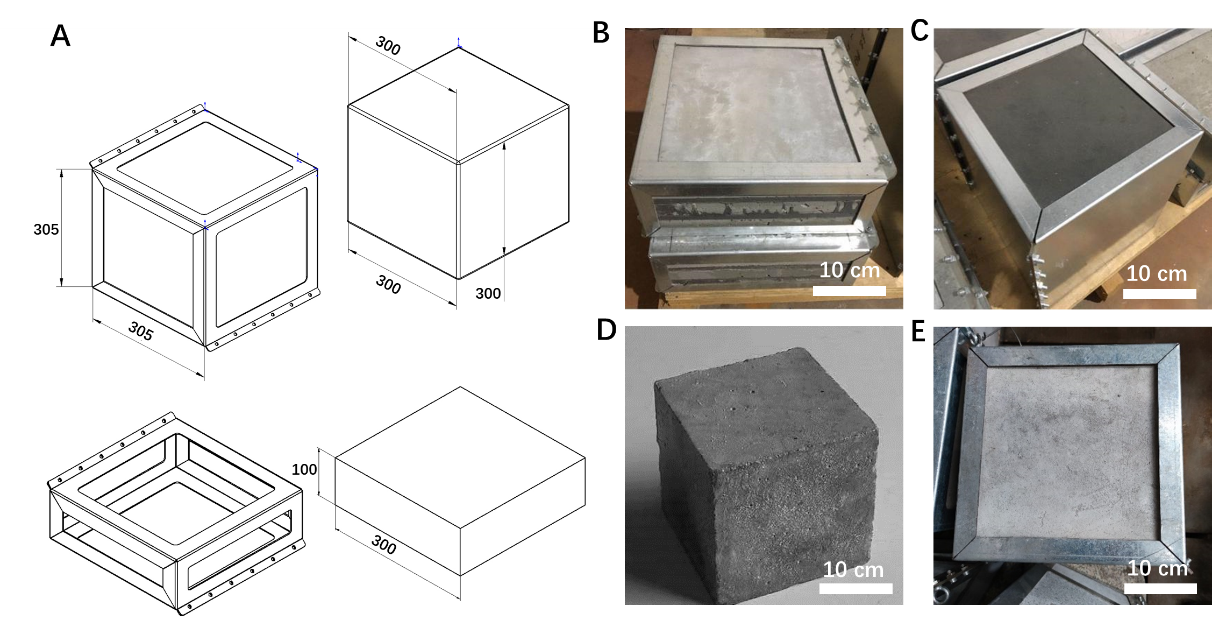
**

**Supplementary Fig. 3.** Schematic diagram of home-made galvanized iron molds for packaging these target materials and digital photos of these packaged target materials. **(A)** Design of two types of home-made galvanized iron molds (305 mm × 305 mm × 305 mm and 305 mm × 305 mm × 105 mm) for packaging these different sized target materials. **(B)** The galvanized iron-packaged nacre-inspired steel-concrete (NISC) target materials (Size: 300 mm × 300 mm × 100 mm). **(C)** The galvanized iron-packaged NISC target materials (Size: 300 mm × 300 mm × 300 mm). **(D)** Pure concrete without galvanized iron frame (Size: 300 mm × 300 mm × 300 mm). **(E)** The galvanized iron-packaged concrete target material (Size: 300 mm × 300 mm × 300 mm).

**
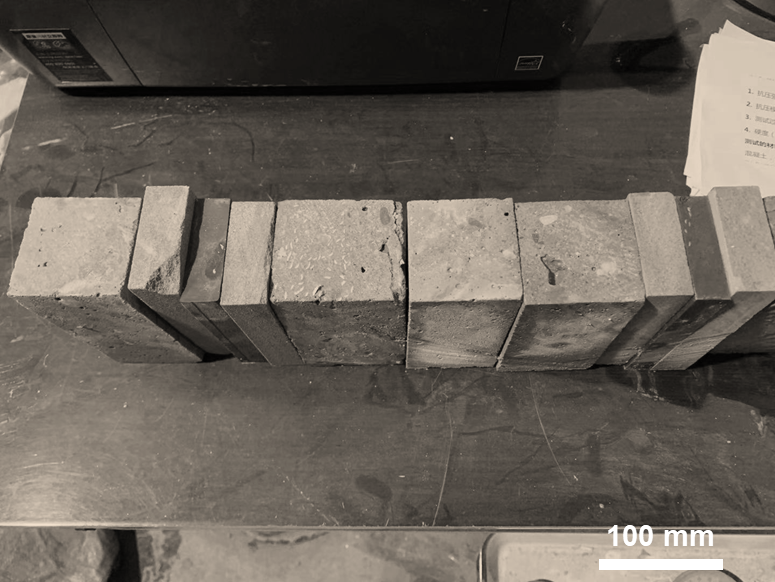
**

**Supplementary Fig. 4.** Digital photo of typical NISC [test](javascript:;) [specimen](javascript:;)s for static mechanical tests (see detailed dimensions in Supplementary Table 1).


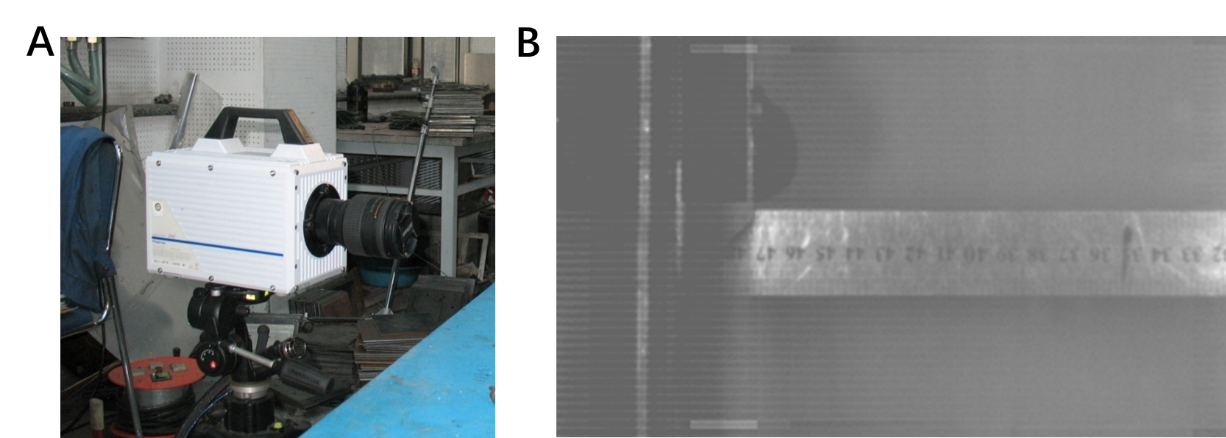


**Supplementary Fig. 5.** Digital photos of Photron SA5 high-speed camera **(A)** and the calibration image **(B)** for recording a striking velocity of a projectile.

**
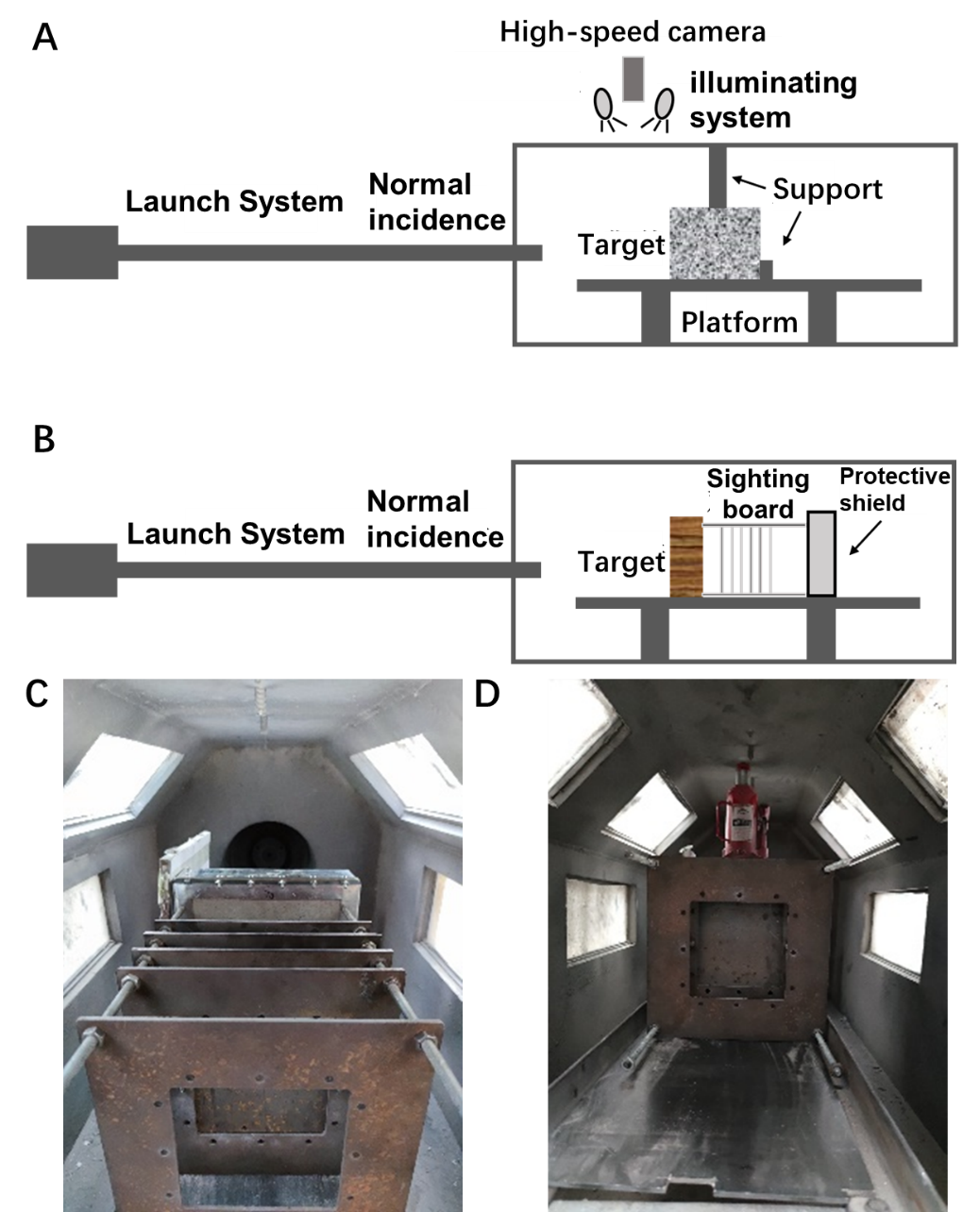
**

**Supplementary Fig. 6**. Schematic diagram and [digital](javascript:;) [photo](javascript:;)s of the experimental support set-up for hypervelocity impact tests. **(A)** Schematic diagram of experimental upper support and rear support for the relatively thick target materials (such as NISC with 300 mm thickness, labeled as NISC-300). **(B)** Schematic diagram of the experimental sighting boards and protective shield for the relatively thin target materials (such as NISC with 100 mm thickness, labeled as NISC-100). (**C, D**) The [digital](javascript:;) [photograph](javascript:;)s of the experimental sighting boards on the experiment platform.

**
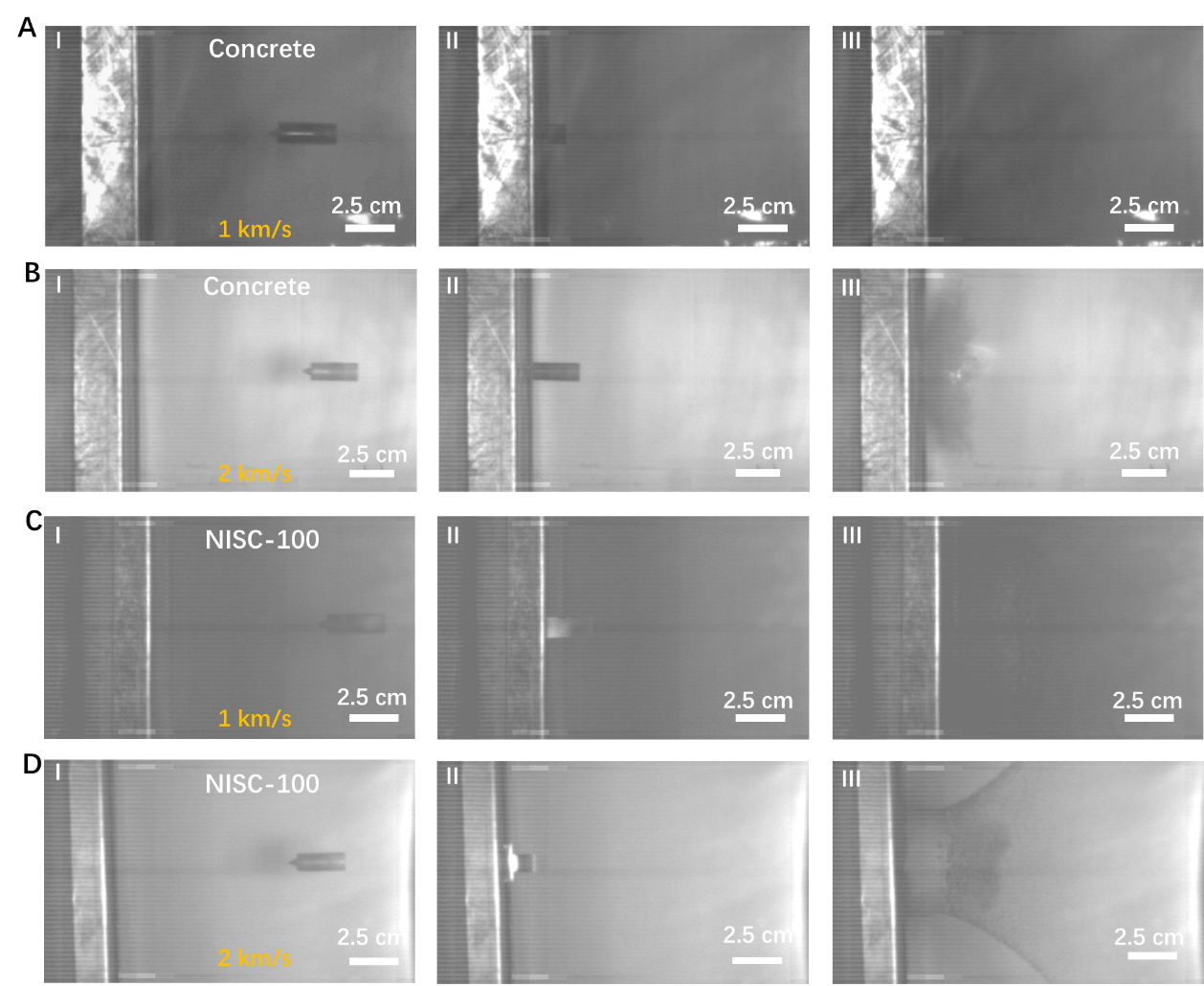
**

**Supplementary Fig. 7.** The [digital](javascript:;) [photo](javascript:;)s for the *in situ* observation of the [impact](javascript:;) [experiment](javascript:;) to typical target material such as pure concrete and NISC-100, recorded by the high-speed camera at different impact speeds. From left to right: I) the projectile is flying at high speed; II) the projectile is impacting the surface of the target; III) the projectile is penetrating into interior of the target, [accompanied](javascript:;) [by](javascript:;) an explosion of debris. **(A)** The [impact](javascript:;) [experiment](javascript:;) process of pure concrete with 100 mm thickness at a striking velocity of 1 km/s. **(B)** The impact process of pure concrete with 100 mm thickness at a striking velocity of 2 km/s. **(C)** The impact process of NISC-100 at a striking velocity of 1 km/s. **(D)** The impact process of NISC-100 at a striking velocity of 2 km/s. Note that the whole detailed [impact](javascript:;) processes are recorded in Supplementary Movies 1 to 3.

**
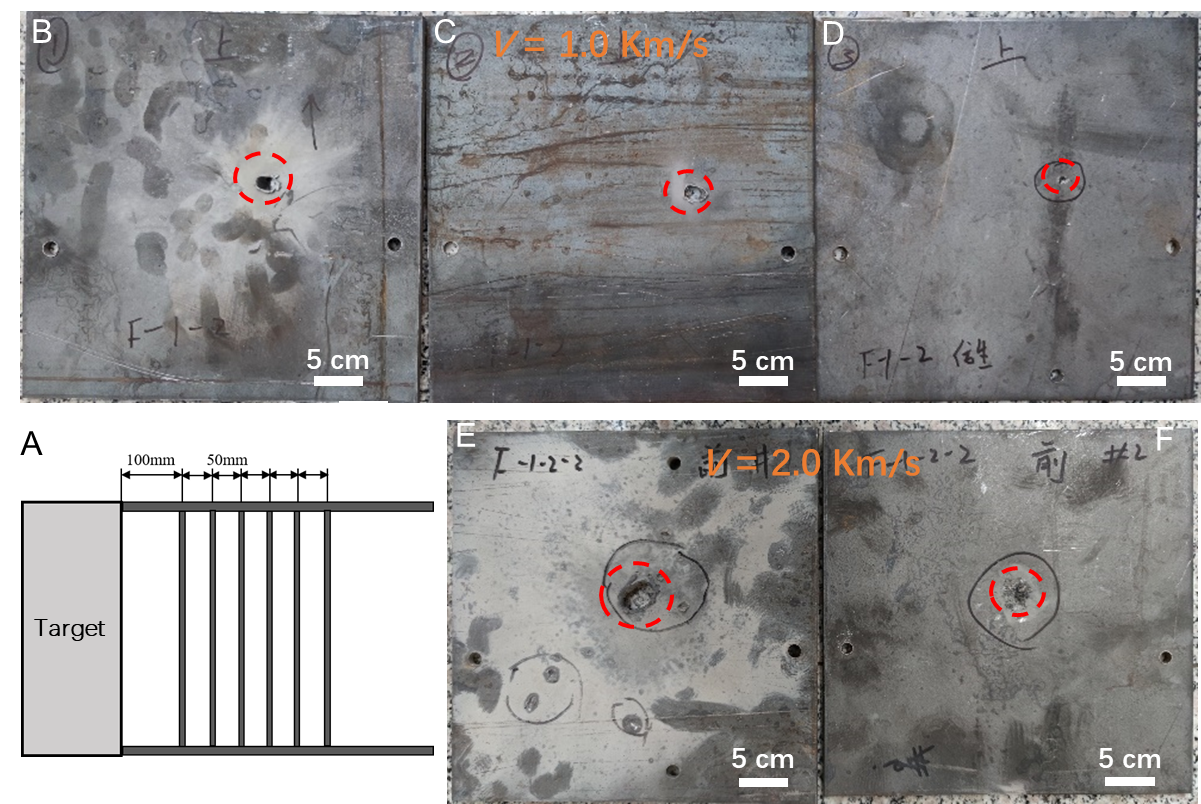
**

**Supplementary Fig. 8.** The morphological observation and size of the bullet holes in the experimental sighting boards, derived from the hypervelocity impact tests of NISC-100 at different striking velocities. **(A)** Schematic diagram of the [overall](javascript:;) [arrangement](javascript:;) of the experimental sighting boards behind a target. **(B-D)** The morphologies and sizes of the bullet holes in the different sighting boards at a striking velocity of 1 km/s. From left to right: the first layer (**B**), the second layer (**C**), and the third layer (**D**). **(E, F)** The morphologies and sizes of the bullet holes in the first (**E**) and second (**F**) layered sighting boards at a striking velocity of 2 km/s, respectively.

**
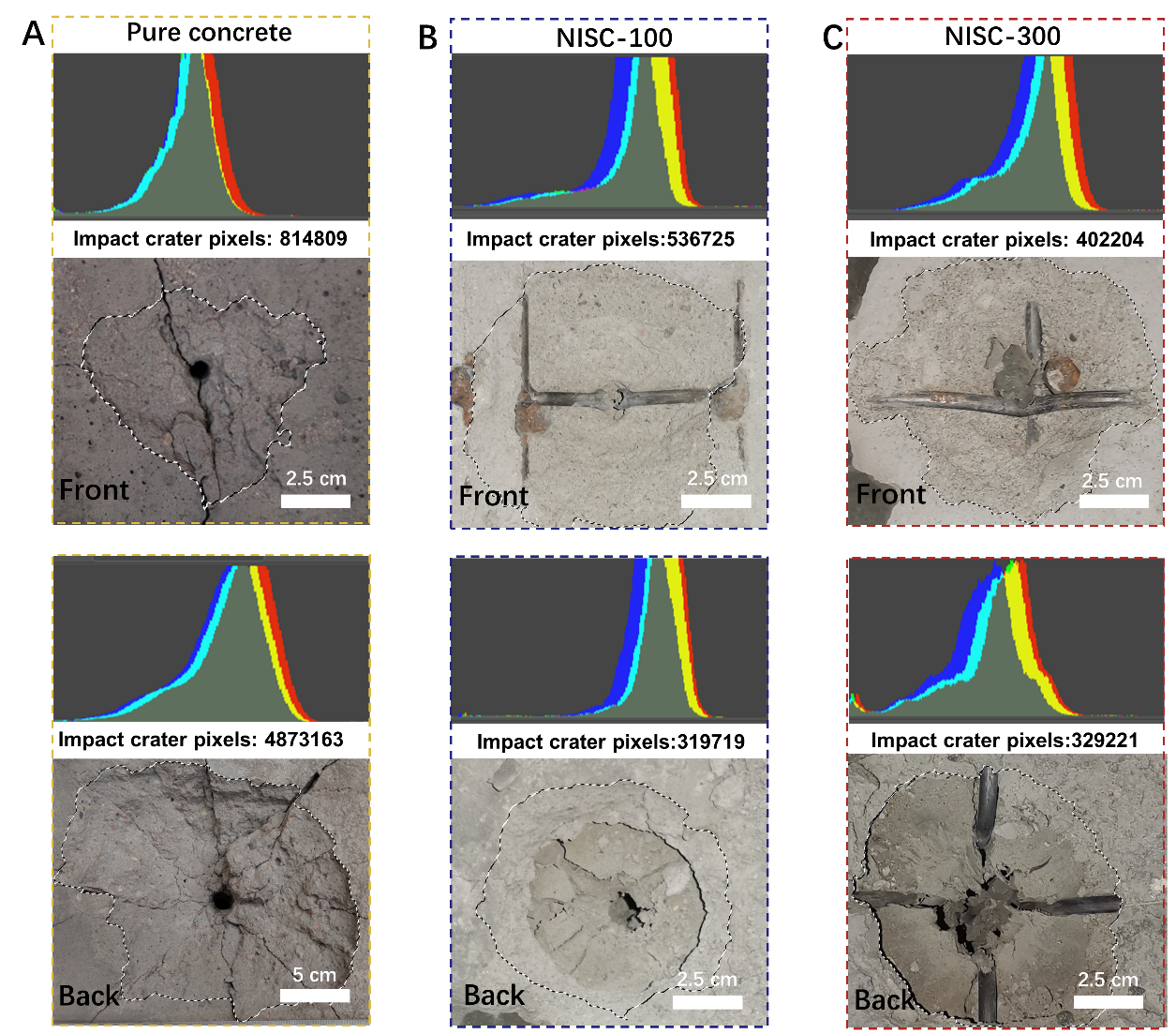
**

**Supplementary Fig. 9.** The analysis of contour areas of the impact craters on the front and back surfaces of typical target materials such as pure concrete (**A**), NISC-100 (**B**), and NISC-300 (**C**)**,** respectively, achieved by [digital](javascript:;) [camera](javascript:;) and Photoshop software. Top: the frontal striking surface of the target material; Bottom: the back surface of the target material.

**Supplementary Table 1.** Summary of the dimensions of the cutting target materials for static mechanical tests.

| Target | [Length](javascript:;) × [Width](javascript:;) × [Height](javascript:;) ([Compression](javascript:;) [test](javascript:;)) | Length × Width × Height  (Bending test) |
| --- | --- | --- |
| Pure concrete | 90 mm × 90 mm × 150 mm | 160 mm × 20 mm × 40 × mm |
| NISC | 90 mm × 90 mm × 150 mm | 160 mm × 20 mm × 40 mm |

**Supplementary Table 2.** Statistical summary of the striking velocities and projectile masses during the hypervelocity impact tests by using a two-stage light gas gun.

| Targets | Set velocity  (km/s) | Projectile mass  (g) | Total projectile mass  (g) | Actual velocity  (km/s) |
| --- | --- | --- | --- | --- |
| Pure concrete | 1 | 5.76 | 9.98 | 1.080 |
| Pure concrete | 2 | 5.76 | 10.16 | 1.937 |
| NISC | 1 | 5.76 | 10.01 | 1.118 |
| NISC | 2 | 5.76 | 10.10 | 1.984 |
